# Applications of Generative Artificial Intelligence for Strabismus Surgery Video-Based Education

**DOI:** 10.1016/j.xops.2026.101144

**Published:** 2026-03-06

**Authors:** Jimmy S. Chen, Marissa Stinnett, Clarissa Camarena, Akshara R. Legala, Elena Flester, Gustavo Wanderer, Jennifer Bu, Skenda Jean Charles, Erika C. Acera, Shira L. Robbins, David B. Granet

**Affiliations:** 1 Viterbi Family Department of Ophthalmology, Shiley Eye Institute, University of California San Diego, La Jolla, California; 2 Department of Ophthalmology, Massachusetts Eye and Ear Infirmary, Harvard Medical School, Boston, Massachusetts

**Keywords:** Artificial intelligence, Patient education, Video-based education, Generative artificial intelligence, Strabismus surgery

## Abstract

**Objective:**

To develop educational artificial intelligence (AI)–generated videos for patients undergoing strabismus surgery and assess patient perceptions of these videos.

**Design:**

Prospective interventional study.

**Subjects:**

Adult patients with strabismus seen at University of California San Diego who were evaluated and subsequently underwent strabismus surgery.

**Methods:**

A pipeline of AI software including Claude Sonnet, Sora, Visla, and Tavus were used to generate a script educating patients on strabismus surgery, create avatars of real ophthalmologists, and AI-generated video clips. Surveys on Google Forms were created to (1) preoperatively assess whether patients could discern whether the videos were AI-generated and educational and (2) postoperatively whether the video remained educational. These videos were disseminated to patients once consent for strabismus surgery was obtained, and surveys were given to patients preoperatively and postoperatively.

**Main Outcome Measures:**

Patient perceptions and educational value of the video were assessed using Likert scales and yes versus no questions, which were averaged or enumerated, respectively. Subjective comments were also thematically assessed.

**Results:**

Thirty patients who underwent strabismus surgery were included in this study. The mean age was 54.0 ± 19.8 years with the majority undergoing surgery for the first time (n = 23 [76.7%]). Patients responded that these videos improved their understanding of strabismus (4.3 ± 0.9), strabismus surgery (4.2 ± 1.1), and eased their concerns (4.5 ± 0.8). Additionally, patients generally did not recognize that the video was AI-generated (4.3 ± 1.1), responded that they would watch more AI-generated videos in the future (4.7 ± 0.7), and would recommend AI-generated content to others (4.7 ± 0.65). When surveyed postoperatively, this sentiment persisted. Subjectively, patients generally emphasized how this video was both educational and reassuring.

**Conclusions:**

In this proof-of-concept study, patients generally felt AI-generated videos for strabismus surgery added educational value. Additionally, most patients perceived the AI-generated content positively. Future studies with larger sample sizes and outcome-based and validated educational endpoints are needed to prospectively evaluate the clinical impact of AI-generated educational content on other conditions and interventions. This approach may open new opportunities to improve patient understanding and ability to contribute to their care.

**Financial Disclosure(s):**

Proprietary or commercial disclosure may be found in the Footnotes and Disclosures at the end of this article.

Strabismus is characterized by ocular misalignment between the 2 eyes and is an important cause of visual and psychosocial impairment among children and adults.^1^ The prevalence of strabismus is estimated to be 4% in the general population. Children with strabismus are at risk for developing amblyopia, and both children and adults with strabismus may experience decreased stereopsis and diplopia. The negative impact of strabismus on quality of life in both children and adults is well-documented in the literature.^1^, ^2^, ^3^, ^4^, ^5^, ^6^, ^7^, ^8^ Specifically, strabismus is associated with increased prevalence of depression, anxiety, and schizophrenia, among other psychiatric diseases.^2^^,^^7^ Patients with strabismus also face social stigmatization, including being perceived as less attractive, intelligent, and competent compared with orthotropic individuals.^8^, ^9^, ^10^

Strabismus surgery is an effective treatment for strabismus and has been shown to improve both functional and psychosocial outcomes in patients,^11^ with 60%–85% successful primary realignment after 1 surgery.^12^, ^13^, ^14^, ^15^, ^16^ Despite the benefits of strabismus surgery, anecdotal evidence among multiple studies have noted long delays between strabismus onset and surgery, particularly in adult patients.^17^^,^^18^ When surveyed about reasons for delays, patients most commonly reported not being offered surgery, fear of surgical complications, perceptions of being too old or unbothered by appearance, and not seeking care as key barriers.^17^^,^^18^ Prior work has also shown that in addition to improved functional outcomes in patients who underwent strabismus surgery, many patients noted improved psychosocial factors including improvements in depression and anxiety, as well as increased confidence.^19^, ^20^, ^21^

While patient education has been proposed to bridge the gap between patient fears and realizing the benefits of strabismus surgery,^22^ there exist few published resources for patient education in strabismus. A systematic review by Frank et al^23^ demonstrated that the majority of educational resources were printouts, with far fewer studies using video content; however, all resources resulted in increased knowledge, decreased anxiety, and improved adherence. More recently, the popularity of social media has resulted in increased educational resources on platforms such as TikTok and Instagram.^24^Additionally, advances in generative artificial intelligence (AI), particularly large language models (LLMs) (such as ChatGPT, Claude, etc.), have transformed patient education within ophthalmology, including question-answering^25^, ^26^, ^27^, ^28^, ^29^ and generating informative handouts.^30^ Within pediatric ophthalmology, the bulk of educational content using LLMs has focused on strabismus,^31^^,^^32^ amblyopia,^33^^,^^34^ and myopia.^35^^,^^36^ With the advent of generative AI for video content, there now exists opportunities for patient education beyond text handouts. However, there is a paucity of literature exploring how video-based generative AI could be used for patient education within strabismus as well as ophthalmology and medicine and its implications.^37^

Our proof-of-concept study aims to address this gap in knowledge by (1) creating an AI-generated educational video for strabismus surgery using a pipeline of generative AI software and (2) assessing patient perceptions of this AI-generated video in a clinical setting.

## Methods

This study was approved by the University of California San Diego Institutional Review Board and adheres to the Declaration of Helsinki. The need for informed consent was waived for this study.

An educational video on strabismus surgery was generated using the following AI-based tools:

Visla, Inc,^38^ Claude Sonnet (Anthropic) and Sora (OpenAI),^39^ and Tavus Inc.^40^ Using zero-shot prompt engineering, Claude Sonnet^41^ was used to generate a script with the goals of (1) defining strabismus, (2) describing strabismus surgery, (3) discussing the risk and benefits of the surgery, and (4) explaining instructions and expectations for postoperative care. Our prompt engineering strategies employed included role play (i.e., act as an expert ophthalmologist), defining the context (i.e., create a short educational video), specific goal-setting (i.e., defining strabismus, its pathophysiology, possible treatments, and importance of adherence), and clarifying the target audience (i.e., for adults). We also iteratively and conversationally improved the script to refine content. The script was reviewed by 3 ophthalmologists (J.S.C., D.B.G., and S.L.R.) for veracity and factual accuracy. We used the Ophthalmic Mutual Insurance Company consent form for strabismus surgery to guide our prompt engineering; an example is shown in Supplemental Figure S1, available at www.ophthalmologyscience.org. Our video script is in Supplemental Figure S2, available at www.ophthalmologyscience.org.

Tavus was then used to generate an avatar, or a realistic-appearing AI-generated video of a real human. Tavus is an online suite of generative AI tools for creating human-like videos including conversations, video generation, and lip syncing.^40^ We trained Tavus to create avatars of 2 pediatric ophthalmologists (D.B.G. and S.L.R.) and subsequently generated a video of both pediatric ophthalmologists reading the script. A video consent was recorded by both ophthalmologists as part of the training process.

Visla, an AI-based video editor,^38^ was subsequently used to create a video of consecutive and realistic-appearing AI-generated clips based on the aforementioned script input. OpenAI’s short video generating software, Sora,^42^ was used to supplement additional AI-generated clips. The avatar videos of the 2 ophthalmologists were also incorporated into the AI-generated clip video such that the AI-generated voices of the ophthalmologists narrated a video interspersed with both avatars and various clips. The strabismus video was approximately 6 minutes in length and is publicly available at:https://www.youtube.com/watch?v=KMnC1yIMrL0.

### Survey Design

Two surveys were created using Google Forms^43^ for data collection. The first survey was designed to be given after the patient watched the AI-generated video at a preoperative visit before the strabismus surgery. This survey collected the following information: limited demographic data (age, ethnicity), history of strabismus surgery, review of written educational handouts before watching the video, as well as various statements assessing patient perceptions of the video including the entertainment and educational utility of the video and whether the video being AI-generated affected the educational experience. These statements were graded using a 1–5 Likert scale, with 1, 3, and 5 equivalent to Strongly Disagree, Neutral, and Strongly Agree, respectively. The second survey was designed to be given postoperatively. This survey again asked about patient perceptions regarding the video, its educational utility, and whether the video saved them from sending a message to their surgeon via the patient portal. All questions in this postoperative survey were asked in a “yes” or “no” style. For both surveys, patients were also provided the option of giving subjective free-text comments. These surveys are provided in Supplementary Figures S3 and S4, available at www.ophthalmologyscience.org.

### Data Collection

**Figure 1:**
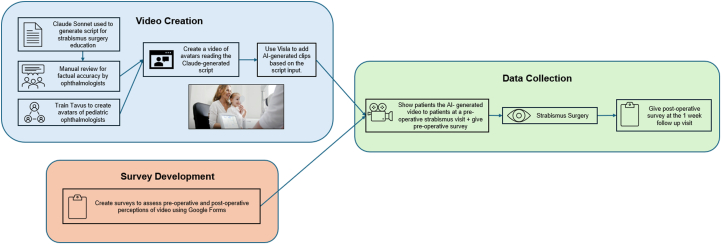
Methods of educational video creation and data collections. AI = artificial intelligence.

Patients seen at the University of California San Diego for an adult surgical strabismus evaluation between March 1, 2025 and July 31, 2025 were recruited for this study. Patients were included in this study if they were (1) over the age of 18, (2) underwent a surgical strabismus evaluation and strabismus surgery, (3) watched the AI-generated educational video preoperatively, and (4) completed the preoperative survey. The AI-generated video was shown at either the initial surgical consultation, where surgical consent was obtained if the patient elected to proceed with surgery, or at the preoperative visit (where the final sensorimotor examination was performed for surgical planning). A folder containing an educational handout on strabismus surgery and information regarding the surgeon was given to each patient as part of protocol. The preoperative Google Forms survey was shown to the patient at either one of these visits. Postoperatively, patients were given a link to the YouTube video for review outside of the office and were recruited to complete the secondary postoperative Google Forms survey at their 1-week postoperative visit. It was not required for patients to watch the video or fill out the second survey postoperatively for inclusion. A diagram of our methodology is shown in Figure 1.

### Data Analysis

All data analysis was performed using R version 4.3.0 (R Foundation for Statistical Computing).^44^ Flesch–Kincaid readability scores were calculated for our script. Responses for demographic and binary survey questions were counted. For all questions using the Likert Scale, mean scores and standard deviation were calculated. Subjective responses were compiled based on theme and sentiment (positive vs. negative).

## Results

Prior to video development, Flesch–Kincaid scores were calculated for our script. The Fleisch Reading Ease score was 70.5, yielding a seventh grade level script. Overall, 30 patients completed both the preoperative survey and underwent strabismus surgery. The mean age was 54.0 ± 19.8 years, and 19 (63.3%) identified as not Hispanic or Latino/a/x. The majority of patients reported English as their first language (n = 25 [83.3%]). Additionally, 23 (76.7%) patients reported this was their first strabismus surgery, and 7 (23.3%) reported that this was at least their second strabismus surgery. The majority of patients watched this video at their preoperative visit (n = 25 [83.3%]). Although all patients received a handout as part of the consent process, 26 (86.7%) confirmed receiving the handout, and 19 (63.3%) confirmed reading the handout prior to watching the video. These data are shown in Table 1.

**Table 1:** Demographics and Surgical Status of Included Patients

Demographic Feature	Mean ± Standard Deviation (Years)
Age	54.0 ± 19.8 years
	Count [n (%)]
Ethnicity	
Hispanic/Latino/a/x	11 (36.7%)
Not Hispanic/Latino/a/x	19 (63.3%)
English as first language	
Yes	25 (83.3%)
No	5 (16.7%)
History of prior strabismus surgery	
Yes	7 (23.3%)
No	23 (76.7%)
Received educational handout prior to watching video	
Yes	26 (86.7%)
No	4 (13.3%)
Read educational handout prior to watching video	
Yes	19 (63.3%)
No	11 (36.7%)
Visit type	
Initial consultation	5 (16.7%)
Preoperative visit	25 (83.3%)
Total	30

**Figure 2:**
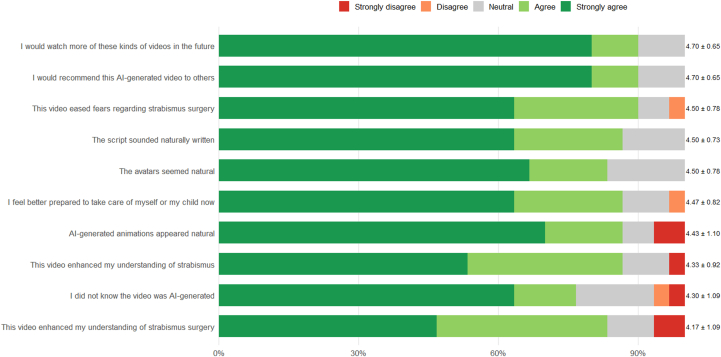
Likert scale plot. Mean and standard deviation Likert scale scores were calculated for questions regarding patient perceptions regarding the educational content of the AI-generated videos as well as perceptions of the script and avatars. AI = artificial intelligence.

Patient sentiments toward the video were very positive when evaluated using the Likert scale (mean ± standard deviation), as shown in Figure 2. Patients generally felt that these videos improved patient understanding of strabismus (4.3 ± 0.9), strabismus surgery (4.2 ± 1.1), eased their fears regarding strabismus surgery (4.5 ± 0.8), and gave them more confidence to take care of themselves or their child (4.5 ± 0.8). Additionally, patients generally did not know that the video was AI-generated (4.3 ± 1.1), and more specifically, felt the animations (4.4 ± 1.1), avatar (4.5 ± 0.8), and script (4.5 ± 0.7) were very natural. Overall, patients strongly felt that they would watch more of these videos in the future (4.7 ± 0.7) and would recommend this kind of AI-generated content to others (4.7 ± 0.7).

Of the 30 patients who completed the survey preoperatively, 27 completed the postoperative survey. The majority of patient respondents postoperatively did not watch the video again (n = 17 [63.0%]). Approximately a quarter of the patients felt postoperatively that the video saved them from sending a message to the physician’s office (n = 6 [22.2%]) and similarly helped with postoperative care (n = 10 [37.0%]). The large majority of patients postoperatively responded that they would still recommend the video to others (n = 25 [92.6%]) and that even after surgery, the AI-generated nature of the video did not negatively affect their perceptions (n = 25 [92.6%]). This data are shown in Table 2.

**Table 2:** Responses to Survey Given Postoperatively

	Count (n [%])
Did you watch the video again since surgery?	
Yes	10 (37.0%)
No	17 (63.0%)
Did this video save you from sending a MyChart message?	
Yes	6 (22.2%)
No	21 (77.8%)
Did watching it again help with you caring for yourself or the patient?	
Yes	10 (37.0%)
No	17 (63.0%)
Would you still recommend the video to others?	
Yes	25 (92.6%)
No	2 (7.4%)
Does knowing that the video is AI-generated affect your perceptions of the video content?	
Yes	2 (7.4%)
No	25 (92.6%)

AI = artificial intelligence.

Comments provided by patients in the surveys preoperatively and postoperatively were reviewed and qualitatively analyzed thematically. The majority of comments focused on the clarity of explanations and improved understanding of strabismus and expectations after surgery, as well as appreciation for more information in general. Two patients also emphasized how they were impressed the content was AI-generated. Patients additionally expressed feedback regarding information they would have appreciated in the video, particularly focused on postoperative recovery (i.e., when they were able to drive, how long eye redness would persist, and expectations for pain improvement). Three patients commented on how one of the ophthalmologist’s avatars appeared unnatural. Two patients also expressed negative sentiments toward the video, one toward the AI-generated content and one finding the handout more useful. These data are shown in Table 3.

**Table 3:** Selected Free-Text Comments on the AI–Based Video from Patients

Themes	Example Comments
Unaware it was AI	“Wouldn’t have known!” “Surprised it was all AI!”
Educational value	“Valuable & I would like to be able to share it with my spouse so he more fully understands strabismus, surgery and approximate post-op & recovery” “Such a good video! Made me feel seen and reinforced the positive postoperative outcomes” “Greatly answers some questions I previously have. AI or not, videos like these makes the surgery less frightening.” “Everything was explained perfectly.” “Just reaffirms what I know mostly, plus a few risks explained more thoroughly.” “Yes, I learned about the improvements in vision postsurgery.” “I support getting as much info about surgery in advance. The video was a helpful source of extra info in an easy to consume format.”
Content patients wished for	“When you can go back to driving, how long after care will take.” “Timeline for post-op pain or recovery or ability to drive.” “How red your eye stays.”
Feedback	“Could be more descriptive of actual surgery.” “Just film your own video if you feel it’s important.” “Dr, Granet looks better than his AI image which could be better. Overall, I think the video is good and very informative.” “Only watched the video once but would be nice to watch again maybe 2 wks postsurgery when recovery is ongoing.” “The handouts were much more helpful to us than was the video.”

AI = artificial intelligence.

## Discussion

In this proof-of-concept study, we demonstrate that an AI-based pipeline can create an educational video on strabismus surgery and that this video adds patient-perceived educational value in a clinical adult strabismus practice. Our study has 3 key findings: (1) multimodal generative AI tools can be integrated at all steps of video creation with human supervision; (2) the AI-generated videos were overall perceived by patients to be educational especially preoperatively; and (3) patients generally had difficulty discerning that the videos were AI-generated and did not feel that this detracted from the educational quality of the video.

Our first key finding is that multimodal generative AI tools can be integrated at all steps of video creation with human supervision. This study adds to the relative dearth of studies published within pediatric ophthalmology overall,^23^ of which only one prior study was video-based. Recent work in generative AI video has focused on avatars for language translation^37^ and generating synthetic medical lecture videos.^45^ Our pipeline utilizes software on the cloud and may reduce the time and effort required to generate a script and video recording. Furthermore, our methodology is scalable, with the majority of manual effort required at the following points: (1) script generation by prompt engineering and manual human review with editing; (2) uploading training videos to Tavus for avatar generation; and (3) stitching of video clips with avatar videos. These steps were manually verified to reduce the rate of hallucinations, customize the video for our institution, and ensure a high quality video. For example, avatars can easily be changed to the patient’s known physician instead of the 2 ophthalmologists in our videos and scripts can be modified manually and via LLM per physician preference. Future work may focus on modifying our script for different reading levels (i.e., for children) and using different languages, as well as for other diseases (amblyopia, glaucoma, macular degeneration). This methodology may be broadly applicable to educational resources across health care in general, including other medical specialties, trainee education, and for training of ancillary staff (i.e., eye pressure measurement, drop application). Most importantly, these videos may rapidly improve access to instructive resources and represent a new frontier in medical education.

Our second key finding is that the AI-generated videos were overall perceived by patients to be educational, particularly preoperatively. In our study, patients expressed strong sentiments that these videos increased their understanding of strabismus and strabismus surgery and highly recommended the videos to other patients both preoperatively and postoperatively. This is consistent with prior evaluations of LLMs demonstrating accurate responses to questions on strabismus.^31^^,^^32^^,^^46^ Specifically, patients found reassurance and expectations of postoperative outcomes to be highly valuable. Interestingly, other patients also had specific questions regarding postoperative recovery (i.e., when to drive, eye redness); thus, patient feedback for these videos will be helpful in improving future iterations. Another reason for our video’s positive perceptions likely lies in its relatively easy comprehensibility, with a seventh grade level script. Our script lies close to the American Medical Association's recommendations for educational materials to be around the sixth grade level based on the average reading skill of American adults at an eighth grade level.^47^ This is in contrast to recent evidence demonstrating that commonly published strabismus resources are often at an eighth to 12th grade level,^48^ as are answers for strabismus questions generated by LLMs.^31^^,^^46^ With prompt engineering, these scripts could be customized for children, a key group that undergoes strabismus surgery, particularly in terms of educating and reducing fear and anxiety.^49^ Interestingly, many patients subjectively did not feel the video improved their ability to take care of themselves or decreased their need to send a message to the physician. Because our study only assessed patient perceptions, future studies assessing patient-centered outcomes including changes in health literacy, postoperative adherence, surgical outcomes, and messages sent are needed.

Our third key finding is that patients generally had difficulty discerning that the videos were AI-generated and did not feel that this detracted from the educational quality of the video. Across the surveys given preoperatively and postoperatively, patients overwhelmingly felt the script, animations, and avatars were natural and undistracting from the educational content, although a few patients did observe unnatural features of one ophthalmologist’s avatar. Prior work evaluating synthetic versus real fundus photographs for retinopathy of prematurity also highlighted expert inability to discern AI-generated content.^50^ Furthermore, several studies in the clinical setting have also implemented LLMs in responding to patient messages, with possibly more empathetic responses compared to humans.^51^^,^^52^ Altogether, these data highlight the potential role of generative AI in medicine ranging from diagnosis, research, shared decision-making, documentation, and patient education. However, use of physician avatars in a malicious or unethical manner presents important risks in eroding public and patient trust in physicians. For example, hallucinations from avatars or uncanny valley effects may both serve to undermine trust in the physician–patient relationship. Similarly, while these avatars are owned by the user (per Tavus guidelines), security breaches have potentially catastrophic consequences. Rigorous regulatory and ethical evaluation of these models will also be important to ensure these models are deployed in a safe, transparent, and equitable manner for patients and clinicians alike.^53^

This study has limitations that may be addressed in future work. First, our study was limited to a small subset of patients at an adult strabismus clinic at a single academic center. Multicenter evaluation with a larger sample size will be necessary to evaluate the generalizability of these findings across different populations and diseases. Second, our video was given in addition to the usual written educational materials as part of routine clinical care. Head-to-head evaluation between AI-generated versus and human-generated educational materials will be important to assess the impact of AI-generated materials. Third, we did not evaluate patient outcomes including adherence rates to surgery instructions, postoperative alignment, or complications as part of this proof-of-concept study. Future work should focus on evaluation of patient outcomes to demonstrate the impact of these interventions.

Overall, a suite of generative AI tools can create a realistic-appearing educational video for strabismus and strabismus surgery that patients generally perceive positively and find educational. However, ethical concerns regarding physician use as avatars need to be addressed, and larger prospective studies need to be performed to assess patient outcomes and generalizability across populations, languages, reading comprehension levels, and diseases. Ongoing collaboration between clinicians, patients, ethicists, and policymakers will be needed to better understand how to most safely and effectively deploy these tools for the betterment of health care delivery.
