# Supplementary material for: Applications of Generative Artificial Intelligence for Strabismus Surgery Video-Based Education
Source: Ophthalmol Sci. 2026 Mar 6;6(5):101144. doi: 10.1016/j.xops.2026.101144 (PMC13084662; doi:10.1016/j.xops.2026.101144)
Supplement: Supplemental Figure S1 [file mmc1.pdf]

Supplemental Figure 1. Guiding Prompt Example for Video Script Generation

Example Prompt

Act as an expert ophthalmologist and create an award-winning script for an AI-generated video on strabismus surgery education for patients. To add to the content please weave in and include the information from this form - it is from the ophthalmic mutual insurance company. Specifically, we would like a script defining strabismus, describing strabismus surgery, a discussion of risk and benefits of the surgery, and instructions and expectations for post operative care. Please maintain a warm, caring tone that speaks directly to those facing strabismus surgery, whether for themselves or their child. **Remember the people watching the video will either be having strabismus surgery or their child will be. Let's win an award for this!**

The OMIC consent is as follows:

Strabismus Surgery

Strabismus is when eyes look in two directions. While one eye is focused straight ahead, your other eye is misaligned---meaning that this eye turns in, out, up, or down. Strabismus can be constant or intermittent (comes and goes).

Here are common and medical names for some types of strabismus:

- Crossed eyes, or esotropia
- Out-turned eyes, or exotropia
- One eye is higher than the other, vertical misalignment or hypertropia
- One eye is lower than the other, vertical misalignment or hypotropia

Strabismus can affect people of all ages. Some children are born with crossed eyes. Other children get strabismus later on. Adults may have had strabismus since they were young or get strabismus later in life because of an illness or injury.

Strabismus may be treated, no matter why it happened or how old you are. Without treatment, strabismus can lead to vision problems such as:

- Loss of binocular vision or stereopsis. This is when your two eyes do not work well enough together to provide depth perception (seeing how far away things are).
- Loss of central vision (ability to see clearly), also known as lazy eye or amblyopia. This occurs in young children.
- Limits in peripheral vision (trouble seeing objects at the side).
- Double vision (seeing two of the same image).

There are 3 common treatments for strabismus: 1) eyeglasses, 2) eyeglasses with a prism (that helps line up how you see images), and 3) surgery. Your doctor will recommend one or more of these treatments based on your type of strabismus. For all treatments, the goals are to:

- Correct problems leading to decreased vision. For children, it is best to start treatment early. This helps prevent amblyopia (lost or decreased central vision).
- Straighten or realign the eyes. This is done by surgery. The surgeon will tighten or loosen eye muscles outside your eye. The surgeon will not work inside your eye or remove the eye from its socket.

The goal of strabismus surgery is to straighten or align both eyes so that they can work better together. Strabismus surgery can also help in other ways:

- Some people are more comfortable with their appearance when their eyes look in the same direction.
- For adults and children, strabismus surgery may improve depth perception and increase field of vision (seeing straight ahead and to the sides)
- After surgery, many patients report that they feel good about themselves, can communicate better with others, have more work options, and are better able to read and drive.

As with all surgery, there are risks (problems that can happen) with strabismus surgery. While the eye surgeon cannot tell you about every risk, here are some of the most common or serious:

- **Surgery may not help strabismus.** Some patients still have some strabismus even after surgery. For instance, your eye may be misaligned the same way or now look in a different direction. You may need prism glasses to help with these problems even after surgery. How much strabismus remains depends on how long you have had strabismus, if you have had strabismus surgery before, how well your body heals, whether you have eye disease, if you take other medicines, and if you have other health problems.
- **Need for more surgery**. Each person's brain needs time to adjust to seeing with the eyes in a new position. It takes at least 2 months to know whether strabismus surgery helped align your eyes. Some people need more than one surgery to correct misalignment.
- **Allergic reaction.** You might be allergic to a medicine or item used during the surgery.
- **Problems from local or general anesthesia.** While anesthesia problems are rare, they can happen. Local anesthesia can cause vision loss, bleeding, or a detached retina (when the retina at the back of your eye pulls away from where was attached). General anesthesia can cause heart or breathing problems, brain damage, and death.

Strabismus surgery can also cause vision and eye problems. These include:

- **Vision loss.** After surgery you can have vision loss from bleeding, a detached retina (when the retina at the back of your eye pulls away from where was attached), or an infection.
- **Double vision** (seeing 2 of the same image). Many people have double vision for at least a while after strabismus surgery. This happens because the brain needs time to adapt to seeing with your eyes in a new position. For young children, double vision often goes away in a few days. For young adults, double vision may last 4 or more weeks. For older adults, it can take weeks or months for double vision to go away. Some people find that their double vision lasts even longer, goes away and comes back, or is always a problem. Many people learn to ignore this second image. Others find it helps to wear prism glasses.

- **Continued misalignment**. Your eyes might not be straight even after surgery.
- **Altered eyelid position**. The upper or lower eyelid of one eye may be at a different height than the other eye.
- **Limited eye movements.** It might hard to move your eye.
- **Scar tissue.** This may make it hard for your eye to fully move in some directions. If you need another strabismus surgery, scar tissue might make it harder for the ophthalmologist to line up your eyes. Your eye could stay red. You might not like how your eye looks.
- **Infection**. Some people get an infection in or around the eye after surgery. Most infections are mild and can be treated with antibiotics. A severe infection could cause vision loss or even loss of the eye.
- **Hemorrhage or bleeding**. While rare, you may have severe bleeding in and around your eye. This could injure your eye or cause vision loss.
- **Temporary eye problems** that last for up to 4 weeks. These can include corneal abrasion (a scratch on the eye's surface) and conjunctivitis (redness on the eye's surface).
- **Eye ache and pain**. Most pain from surgery goes away in 1 or 2 days. Call your eye surgeon right away if you have severe pain or if your pain lasts for 3 or more days.
