# Supplementary material for: Applications of Generative Artificial Intelligence for Strabismus Surgery Video-Based Education
Source: Ophthalmol Sci. 2026 Mar 6;6(5):101144. doi: 10.1016/j.xops.2026.101144 (PMC13084662; doi:10.1016/j.xops.2026.101144)
Supplement: Supplemental Figure S2 [file mmc2.pdf]

Supplemental Figure 2. Video Script.

Hello there! I'm Dr. David Granet. And I'm Doctor Shira Robbins. We're here to guide you through a journey that we have devoted our lives to… strabismus surgery. Whether you're a parent whose child needs this surgery, or an adult considering it for yourself, you're not just in the right place; you're with a team that truly cares.
First, let's understand what we're dealing with. Strabismus is when your eyes look in two different directions. While one eye focuses straight ahead, the other turns in… out… up… or down.
In medical terms, we call these

- Crossed eyes, or eesotropia, when the eye turns in.
- Out-turned eyes, or exotropia, when it turns out.
- One eye higher… hypertropia… or lower… hypotropia… than the other.

For some it's always there. For others, it comes and goes. Some have eyes that are off side to side. up and down. And tilted… Strabismus can affect anyone, at any age despite coming in so many different "flavors".

Now, you might be wondering, "Does it matter?" The answer is yes, and here's why. Without treatment, strabismus can lead to

- Loss of depth perception such as trouble judging distances.
- Amblyopia, or what lay people call "lazy eye" in children, where one eye's vision weakens from lack of use.
- Limits in peripheral or side vision.
- Double vision or seeing two of things.

But here's the good news. We have powerful tools to help! There are three main treatments:
First, Eyeglasses. Sometimes they're all we need!
Second, Prism glasses, which help line up what each eye sees.
Third, Surgery or injections when we need to do a bit more.
Our goals? To preserve vision, especially in kids, and to get those eyes working as a team again.

Now, let's talk about strabismus surgery. It's not as scary as it might sound. We're not going inside your eye or—as some think—removing it. Instead, we work on the muscles outside your eye, like fine-tuning a car's steering or getting the headlights aligned. Generally, we make a small incision through the white covering of the eye and can get to the muscles from there. We either tighten or loosen some of the muscles depending on your eye's misalignment.
By tightening or loosening these muscles, we help your eye point in the right direction. It's like choreographing a dance. We're helping your eyes to move in sync. And the benefits? They can be life-changing.

- Your eyes look aligned, which most find an advantage.
- Better depth perception.
- Wider field of single vision.
- And often, a big boost in self-confidence….

Adults may communicate better via eye contact, been shown to have more work options, and may even drive more safely. Kids? They develop better 3D vision, may coordinate better overall, and face less teasing. It also helps with amblyopia.

Now, we know the word "surgery" can be scary. As your guide and partner in medical care, we promise to be fully transparent. There are risks, but most are rare, and knowing about them helps us navigate safely. Despite our extensive and deep experience performing surgery, developing new procedures and teaching surgery around the world, things can still happen that aren't exactly what we want.

First, understand that surgery might not fix everything. Some patients still have some misalignment. Your eye might look better but not perfect, or it could even turn a different way. About 15-20% of people—that's 1 in 5 to 1 in 7—need another surgery later.

There's a small risk of infection, about 1 in 900 surgeries. Most are minor, like a small cut getting infected, and are easily treated. We give you antibiotic drops to prevent this. Very rarely—about 1 in 11,000 to 13,700 cases—a serious eye infection called endophthalmitis can occur... Keeping away from dirt by avoiding things like gardening can help….

There's also a tiny risk of eye damage:

- Rarely surgeries have a complication that could affect vision, like a small tear in the eye wall.
- But here's the reassuring part: it is very rare to have poorer vision six months after surgery. Your eye's healing power is amazing…

Other possible impacts:

- Double vision, usually temporary as your brain adjusts.
- Limited eye movement.
- Rarely scar tissue… which might affect future surgeries.
- Eye aches and pain… usually gone in a few days.
- Rarely… severe bleeding or problems from anesthesia…

For kids, their resilient plastic brains often adapt quickly. Adults, especially if you've had strabismus for a longtime, might need more time. Some find prism glasses help during their adjustment.

Now, let's talk about making this journey as smooth as possible. On surgery day, you're in expert hands.

- Anesthesiologists work to ensure comfort and safety.
- We work using special glasses with magnifiers attached for precision.
- We also keep your eye moist to prevent scratches…

After surgery, you become the hero! Your actions dramatically reduce risks:

- Use eye drops and ointments—they're healing helpers!
- Apply cool compresses— they're like a mini spa treatment.
- Rest is your superpower—give your eyes a break.
- No swimming or contact lenses for a while.

For kids… make recovery fun. You can try:

- Eye drops after a favorite show.
- Special stuffed animals to remind them not to rub.
- Ice cream—cold treats reduce swelling…

Adults… take a week off work, especially screen-heavy jobs... And no driving until we give the green light… If you work in an area of dirt and dust or in a hospital/medical area you may need longer time off work.

We know we've covered a lot. Surgery, risks, recovery—it can feel overwhelming. But here's what we want you to hold onto.

You're embarking on something profound. Whether you're five or fifty-five, this surgery isn't just about aligning eyes; it's about aligning your world. A world where depth pops, where words behave on a page, where one image replaces two. A world seen fully, through eyes that work beautifully in harmony.
The risks are real but rare. The benefits? They're life-altering. By understanding each step and actively participating, you're not just a patient. You're a partner and a hero in your own health story…

Parents, we see your fierce love. Your child's resilience will amaze you. Adults, whether this is a long-time issue or something new, it's never too late. Many wish they'd done this years ago. Please know we care and you are in the right place.

So, whether it's you or your child facing this, remember: This isn't just about straightening eyes. It's about seeing straight—in every sense. It's about moving forward, into a world that's clearer, deeper, and more connected.

I'm Dr. David Granet. And I'm Dr. Shira Robbins. It's been an honor to be a part of your journey. Your more aligned vision awaits. No matter what… we and the entire Shiley Eye Institute and UCSD Health are a part of your team as we go through this together, every step of the way.
