# Supplementary material for: Applications of Generative Artificial Intelligence for Strabismus Surgery Video-Based Education
Source: Ophthalmol Sci. 2026 Mar 6;6(5):101144. doi: 10.1016/j.xops.2026.101144 (PMC13084662; doi:10.1016/j.xops.2026.101144)
Supplement: Supplemental Figure S3 [file mmc3.pdf]

Improving Patient Education on Post-Operative Care After Strabismus Surgery

We are conducting a study to improve care after strabismus surgery. You have been selected to participate in this study because you or a loved one of yours (i.e. child < 18 years of age) was recommended to have strabismus surgery. We have created an educational video in hopes to answer your questions on surgery, how to take care of you or your loved one afterwards, and address your worries and concerns.

All of the materials in this study have been thoroughly reviewed by qualified strabismus surgeons to ensure thoroughness and comprehensibility. Our goal is to understand how different education materials impact patient communication. Your feedback is crucial in helping us assess and improve the quality of patient education. This study should take 5 minutes or less. We appreciate your time and cooperation with this study.

Principal investigators:
Jimmy Chen, MD; Shira Robbins, MD David Granet, MD (UCSD pediatric ophthalmology & strabismus service)

* Indicates required question

1. Please fill out these questions from the perspective of the patient undergoing surgery (i.e. if your child is undergoing surgery, fill out demographic information for them). We will not be collecting any other personal information besides the information requested in this survey. *

   How old are you or your child in years? (Write the number only)

   ____

2. What is you or your child's gender? *

   Mark only one oval.

   - ( ) Male
   - ( ) Female
   - ( ) Non-Binary
   - ( ) Prefer not to answer

3. What is you or your child's race? *

Mark only one oval.

- White
- Black
- Asian / Pacific Islander
- Hispanic
- African American
- American Indian / Native American
- Other

4. What is you or your child's etnicity? *

Mark only one oval.

- Hispanic or Latino/a/x
- Non-Hispanice or Non-Latino/a/x

5. Is English your first language? *

Mark only one oval.

- Yes
- No

6. Have you or your child ever had strabismus surgery? *

Mark only one oval.

- Yes
- No

7. If you answered yes to the above, how long ago?

____

8. Did you receive a surgical education handout in the clinic? *

Mark only one oval.

- ( ) Yes
- ( ) No

9. Did you read the surgical handout prior to watching this video? *

Mark only one oval.

- ( ) Yes
- ( ) No

10. What visit is this for you? *

Mark only one oval.

- ( ) The Surgical Consultation (i.e. signing up for surgery)
- ( ) Pre-operative visit (i.e. already signed up, just getting measurements)
- ( ) Post-operative (i.e. after surgery)
- ( ) Other

11. Have you filled out this survey before? *

Mark only one oval.

- ( ) Yes
- ( ) No

12. For the following statements related to the AI-generated video (yes it is AI generated!), please rank your feelings towards the following statements: *

I would not have known the video was AI-generated unless you told me.

Mark only one oval.

	1	2	3	4	5	
Stro	◯	◯	◯	◯	◯	Strongly agree

13. I feel the AI-generated video enhanced my understanding of strabismus. *

Mark only one oval.

	1	2	3	4	5	
Stro	◯	◯	◯	◯	◯	Strongly Agree

14. I feel the AI-generated video enhanced my understanding of strabismus surgery. *

Mark only one oval.

	1	2	3	4	5	
Stro	◯	◯	◯	◯	◯	Strongly Agree

15. I feel more at ease at the thought of strabismus surgery after watching this video. *

Mark only one oval.

	1	2	3	4	5	
Stro	◯	◯	◯	◯	◯	Strongly Agree

16. After watching this video, I feel better prepared to take care of myself (or my child) following strabismus surgery. *

Mark only one oval.

	1	2	3	4	5	
Stro	◯	◯	◯	◯	◯	Strongly Agree

17. I would recommend these types of educational AI-generated videos to family members and friends undergoing a similar surgery. *

Mark only one oval.

	1	2	3	4	5	
Stro	◯	◯	◯	◯	◯	Strongly Agree

18. I would like to watch more of these kinds of videos for any future surgeries or eye conditions I or my loved ones may have in the future. *

Mark only one oval.

	1	2	3	4	5	
Stro	◯	◯	◯	◯	◯	Strongly Agree

19. The AI-generated animations appeared natural and did not take away from the video experience. *

Mark only one oval.

	1	2	3	4	5	
Stro	◯	◯	◯	◯	◯	Strongly agree

20. The AI-generated avatars of Dr. Granet and Dr. Robbins and voices appeared natural and did not take away from the video experience. *

Mark only one oval.

	1	2	3	4	5	
Stro	◯	◯	◯	◯	◯	Strongly Agree

21. The video script (not regarding the voice delivery of the script) sounded naturally written. *

Mark only one oval.

	1	2	3	4	5	
Stro	◯	◯	◯	◯	◯	Strongly Agree

22. Did you have questions answered by video? If so, what? *

23. Were there any aspects of the recovery process that were not covered in the video that you feel should be? *

24. Any other comments regarding this video?

This content is neither created nor endorsed by Google.

Google Forms
