# Supplementary material for: Applications of Generative Artificial Intelligence for Strabismus Surgery Video-Based Education
Source: Ophthalmol Sci. 2026 Mar 6;6(5):101144. doi: 10.1016/j.xops.2026.101144 (PMC13084662; doi:10.1016/j.xops.2026.101144)
Supplement: Supplemental Figure S4 [file mmc4.pdf]

Post-operative Strabismus Surgery Video Survey

We are conducting a study to improve care after strabismus surgery. You have been selected to participate in this study because you or a loved one of yours (i.e. child < 18 years of age) already had strabismus surgery. We have created an educational video in hopes to answer your questions on surgery, how to take care of you or your loved one afterwards, and address your worries and concerns.

All of the materials in this study have been thoroughly reviewed by qualified strabismus surgeons to ensure thoroughness and comprehensibility. Our goal is to understand how different education materials impact patient communication. Your feedback is crucial in helping us assess and improve the quality of patient education. This study should take 2 minutes or less. We appreciate your time and cooperation with this study.

Principal investigators:
Jimmy Chen, MD; Shira Robbins, MD David Granet, MD (UCSD pediatric ophthalmology & strabismus service)

* Indicates required question

1. Did you watch the video again since having surgery? *

Mark only one oval.

- Yes
- No

2. Did watching the video again save you from sending a MyChart message? *

Mark only one oval.

- Yes
- No

3. Did watching the video after surgery help your care for yourself or your child (whoever had surgery)?

   Mark only one oval.

   - Yes
   - No

4. Would you recommend this video to other patients having strabismus surgery? *

   Mark only one oval.

   - Yes
   - No

5. Does knowing that the video is AI-generated affect your perceptions of the video content? *

   Mark only one oval.

   - Yes
   - No

6. Any comments?

   ____
   ____
   ____
   ____
   ____

This content is neither created nor endorsed by Google.

Google Forms
